# Lipiodol nanoemulsions stabilized with polyglycerol-polycaprolactone block copolymers for theranostic applications

**DOI:** 10.1186/s40824-017-0108-4

**Published:** 2017-10-17

**Authors:** Trang Huyen Le Kim, Hwiseok Jun, Jin Ho Kim, Keunchil Park, Jee Seon Kim, Yoon Sung Nam

**Affiliations:** 1 Department of Materials Science and Engineering, Korea Advanced Institute of Science and Technology, 291 Daehak-ro, Yuseong-gu, Daejeon, 34141 Republic of Korea; 2 Samsung Medical Center, Samsung Biomedical Research Institute, Irwon-dong, Gangnam-gu, Seoul, 06351 Republic of Korea; 3 Samsung Biomedical Research Institute and Division of Hematology and Oncology, Department of Medicine, Samsung Medical Center, Sungkyunkwan University School of Medicine, Irwon-dong, Gangnam-gu, Seoul, 06351 Republic of Korea; 4 Department of Materials Science and Engineering and KAIST Institute for the NanoCentury, Korea Advanced Institute of Science and Technology, 291 Daehak-ro, Yuseong-gu, Daejeon, 34141 Republic of Korea

**Keywords:** Polyglycerol, Amphiphilic copolymers, Nanoemulsions, Lipiodol, Paclitaxel, Cancer

## Abstract

**Background:**

Polyglycerol is an attractive hydrophilic building block of amphiphilic copolymers for biomedical and pharmaceutical applications due to its biocompatibility, facile chemical modification, and anti-fouling activity. Herein we introduce theranostic nanoemulsions incorporating anti-cancer therapeutic and contrast agents using linear polyglycerol-poly(ε-caprolactone) diblock copolymers (PG-*b*-PCL). Lipiodol is used as a core oil that dissolves paclitaxel and serves as a contrast agent for computer tomography (CT).

**Methods:**

PG-*b*-PCL is synthesized by three-step processes: polymerization of ethoxyethyl glycerol ether; ring-opening polymerization of ε-caprolactone; and deprotection of the PEEGE block. In vitro cytotoxicity of the polyglycerolated lipiodol nanoemulsions is demonstrated using HeLa ovarian cancer cells. The applicability of the prepared nanoemulsions as a contrast agent for CT imaging is also evaluated using micro-CT.

**Results:**

Three compositions of PG-*b*-PCL with different block lengths are synthesized to prepare nanoemulsions. The polyglycerolated lipiodol nanoemulsions exhibit excellent anti-cancer activities, while placebo nanoemulsions have no significant cytotoxicity under the same condition. Micro-CT imaging of the nanoemulsions confirms the ability of nanoemulsions as a contrast agent.

**Conclusions:**

This study suggests that PG-*b*-PCL is a promising polymeric emulsifier for effective stabilization and surface functionalization of drug delivery nanocarriers for therapeutic and imaging agents.

**Electronic supplementary material:**

The online version of this article (10.1186/s40824-017-0108-4) contains supplementary material, which is available to authorized users.

## Background

Recently, various amphiphilic copolymers have been studied for drug delivery with aiming to enhancing the therapeutic effects of drugs through long-term blood circulation and facile surface modification [1–3]. Polyethylene glycol (PEG) has been extensively employed as the hydrophilic, non-sticky corona of drug delivery nanocarriers because PEG efficiently suppresses non-specific protein adsorption and colloidal aggregation in the blood stream [4]. However, recent studies showed that a long PEG chain can effectively hinder the cellular uptake of nanoparticles and the following endosomal escape partially owing to its neutrality or lack of responsiveness to environmental changes [5–7]. In addition, although cell adhesion molecules are generally introduced at the end of PEG exposed to the surface, the number of molecules that can be conjugated is very limited, and relatively large, hydrophobic targeting molecules can negatively affect the self-assembly and surface properties of nanocarriers [8–10]. Therefore, alternative hydrophilic polymers have been explored to overcome the limitations of PEG.

Polyglycerol (PG) has received increasing attention as a new hydrophilic polymer due to its unique chemical properties [11–14]. These include a large number of hydroxyl groups, dendritic structures [15], and biocompatibility [16]. PG was also co-polymerized with poly(ε-caprolactone) (PCL) [17], PCL–polycitric [18], and PEG [19] to enhance the intracellular delivery of anti-cancer drugs [20]. In comparison to PEG, the hydroxyl groups on each monomeric unit of PG can help increase the hydrophilic properties of the nanoparticle surface, thereby increasing the dispersion stability of nanocarriers in the presence of serum proteins, and provide multiple sites for the conjugation of targeting molecules.

In this work, we employed a linear PG-PCL diblock copolymer (PG-*b*-PCL) as an emulsifier to stabilize lipiodol nanoemulsions and functionalize the PG block with cancer cell-targeting molecules. Lipiodol, an iodinated derivative of poppy seed oil with a high iodine content (30–40 wt-%) [21], has been used as a clinically approved computer tomography (CT) contrast-enhancing agent and has been used for trans-arterial chemoembolization for hepatocellular carcinoma [22]. Recently, theranostic nanocarriers have been vigorously investigated for the co-delivery of imaging and therapeutic agents to tumor tissues, enabling to identify the precise location of tumors in a non-invasive manner and provide evidences to determine a suitable treatment regimen [23]. Our previous studies demonstrated that PEG-PCL diblock copolymers can form a semi-solid interphase between oil and water, producing robust oil-in-water emulsions [24–27]. The present work extended the same concept of emulsion stabilization to PG-*b*-PCL with lipiodol as a core oil of the nanoemulsions dissolving paclitaxel [28–30]. The cytotoxicity and cellular uptake of the prepared nanoemulsions were investigated to demonstrate the feasibility of lipiodol nanoemulsions stabilized with PG-*b*-PCL as a delivery carrier for anti-cancer drugs.

## Methods

### Materials

Ethyl vinyl ether (99%), glycidol (96%), *p*-toluene sulfonic acid monohydrate (TsOH, 98%), oxalic acid (98%), potassium *tert-*butoxide (>98%), ɛ-caprolactone (97%), tin(II) 2-ethylhexanoate (92.5–100%), 3-aminophenylboronic acid hydrochloride (3-APBA, 98%), folic acid (97%), *N*-(3-dimethylaminopropyl)-*N*′-ethylcarbodiimidehydrochloride (EDC, 98%), *N*-hydroxysuccinimide (NHS), and 4′,6-diamidino-2-phenylindole (DAPI), were purchased from Sigma-Aldrich (St. Louis, Mo, USA). Lipiodol was purchased from Guerbet (Aulnay-sous-Bois, France), and paclitaxel (>97%) from Wako Pure Chemical Industries, Ltd. (Osaka, Japan). Human cervical epithelial carcinoma (HeLa) cells were purchased from the Korean Cell Line Bank (Seoul, Republic of Korea). A Cell-Counting Kit-8 (CCK-8) was obtained from Dojindo Laboratories (Kumamoto, Japan). Dulbecco’s modified Eagle’s medium (DMEM), phosphate buffered saline (PBS), and fetal bovine serum (FBS) were purchased from Invitrogen (Carlsbad, CA, USA).

### Synthesis of linear PG*-b-*PCL

Ethoxyethyl glycerol ether (EEGE) was synthesized from glycidol and ethyl vinyl ether using TsOH as a catalyst at room temperature for 3 h. The product was washed with saturated NaHCO_3_ and then isolated using ethyl acetate and dried using MgSO_4_, followed by distillation at 64 °C in vacuum. Poly(ethoxyethyl glycerol ether) (PEEGE) was synthesized through polymerization of EEGE in THF at 65 °C for 24 h using potassium *tert*-butoxide as an initiator. The produce was precipitated in cold methanol, and then the solvent was evaporated at 30 °C. The synthesized PEEGE was isolated using benzene and dried in vacuum for 24 h. Linear PEEGE-*b*-PCL was synthesized by the ring opening polymerization of ɛ-caprolactone at 125 °C for 20 h using PEEGE and stannous octanoate as an initiator and a catalyst, respectively. The synthesized polymer was dissolved in methylene chloride and precipitated in cold hexane. The de-protection of PEEGE-*b*-PCL was carried out to obtain PG-*b*-PCL by mixing the PEEGE-*b*-PCL solution in acetone with an aqueous solution of oxalic acid. The mixture solution was magnetically stirred for 5 h at room temperature. The mixture was then dialyzed against deionized water through a Spectra/Por membrane for 24 h. The final polymer product was dried by rotary evaporation.

### Characterization of PG*-b-*PCL

Gel permeation chromatography (GPC) analysis was performed using Agilent high performance liquid chromatography (HPLC) 110 series (Agilent Technologies, Palo Alto, CA, USA). Three PL gel columns (300 × 7.5 mm, pore sizes = 10^3^, 10^4^, and 10^5^Å) and a refractive index detector were used. Tetrahydrofuran was used as an isocratic mobile phase at a flow rate of 1.0 mL min^−1^. The molecular weight of the polymers was obtained through calibration against monodispersed polystyrene standards (Polysciences, Inc., Warrington, PA, USA). ^1^H Nuclear magnetic resonance (NMR) analysis was performed at 25 °C using a Bruker NMR (300 MHz) using CDCl_3_ as a solvent. Chemical shifts were measured in parts per million (ppm) using tetramethylsilane as an internal reference. Differential scanning calorimetry (DSC) analysis was performed using DSC 204 F1 Phoenix (Netzsch- Gerätebau GmbH, Selb, Germany). Heating and cooling scans were run between −50 and 200 °C at a scan rate of 10 °C min^−1^.

### Preparation of Lipiodol Nanoemulsions

Various preparation conditions for placebo nanoemulsions (denoted as ‘bNEs’) and paclitaxel-incorporating nanoemulsions (denoted as ‘pNEs’) were investigated. Briefly, the pre-determined amounts of PG*-b-*PCL, lipiodol, and paclitaxel were dissolved completely in acetone by sonication for 10 min. The solutions were then added dropwisely into 10 mL deionized water with vigorous magnetic stirring or homogenization. Various stirring/homogenizing speeds (500–10,000 rpm) and temperatures (25–50 °C) were examined. The mixtures were then stirred overnight for solvent evaporation. The produced nanoemulsions were stored at 4 °C until use.

### Characterization of Lipiodol Nanoemulsions

The hydrodynamic size distribution of the nanoemulsions was determined by dynamic laser light scattering (DLS) using a particle size analyzer ELSZ-1000 (Otsuka Electronics Co., Ltd., Osaka, Japan). The morphology of the nanonemulsions was observed via transmission electron microscopy (TEM) (JEM-3010, JEOL, Japan). TEM specimen was prepared by mixing 10 μL of the nanoemulsions with 10 μL of 2% uranyl acetate for negative staining. The mixture was dropped on a carbon-coated copper grid (Ted Pella, Inc., Redding, CA, USA), followed by incubation at room temperature for 5 min. The drop was removed using a filter paper, and the grids were dried in air.

### Determination of loading yield and efficiency

The nanoemulsions (500 μL) were freeze-dried and dissolved in 1.5 mL ethanol with sonication for 1 h. The solution was filtered through a 0.45 μm syringe filter, and the amount of lipiodol was determined at 260 nm using UV-visible absorption spectrophotometry (UV-1601, Shimadzu, Kyoto, Japan). As for the loading yield and efficiency of paclitaxel, the nanoemulsions (1 mL) were freeze-dried and dissolved in 1 mL acetonitrile with sonication for 1 h. The solution was filtered through a 0.45 μm syringe filter, paclitaxel in the filtrate was determined using reversed phase HPLC (Agilent 1100 series, Agilent Technologies, Palo Alto, USA) equipped with a Waters Spherisorb ODS2 column (C18, 5 μm, 4.6 mm × 250 mm). Acetonitrile was used as an isocratic mobile phase at a rate of 1 mL min^−1^. The absorption of the eluted solution was determined at 227 nm using a standard curve of paclitaxel in acetonitrile.

### Conjugation of folic acids to Nanoemulsions

Folic acid (FA, 1 mg) was dissolved in *N*,*N*-dimethylformamide (DMF, 5 mL) at room temperature with magnetic stirring for 20 min. EDC (440 mg) and NHS (260 mg) was added to the solution, and then the solution was magnetically stirred on ice for 1 h. Then, 3-APBA (411 mg) in DMF (2 mL) was then mixed with the FA solution followed by stirring in an ice bath for 1 h and at room temperature for 24 h. The solution was then added dropwisely into deionized water (50 mL). Yellow precipitates were gathered by filtration and then washed several times with acetone. The folate boronic acid (FBA) powder was dried under vacuum overnight, dissolved in DMSO, and then diluted by deionized water to the final concentration of 20 μg mL^−1^. The FBA solution (5 mL) was mixed with pNEs (1 mL) by magnetic stirring at room temperature for 24 h for esterification, and then non-conjugated FBA were removed by dialysis against deionized water through a Spectra/Por membrane (MWCO = 12 kDa) for 12 h. To determine the degree of FA substitution, the prepared FA-conjugated pNEs (denoted as ‘fpNEs’) were freeze-dried and then re-dissolved in DMSO by sonication for 15 min. The UV absorbance of folates was measured at 363 nm, and the number of FA conjugated to the nanoemulsions was calculated using a calibration curve of FA in DMSO.

### Serum stability and in vitro release

The serum stability of pNEs was determined by incubating the nanoemulsions in PBS containing 10% FBS at 37 °C. The particle size distribution of the nanoemulsions were determined using DLS. The In vitro release profile of paclitaxel was obtained by incubating 20 mL of the nanoemulsions (0.3 mg mL^−1^ of paclitaxel) in a Spectra/Por dialysis bag (MWCO = 12 kDa) placed in 100 mL of PBS containing various percentages of methanol. At pre-determined time intervals, a 1 mL aliquot of the medium was taken, freeze-dried, and dissolved in acetonitrile to determine the concentration of paclitaxel using reversed phase high performance liquid chromatography (RP-HPLC).

### Micro-CT analysis

HeLa cells were seeded onto a 100 mm dish at a density of 10^7^ cells per dish, followed by incubation in DMEM at 37 °C for 24 h. The medium was then replaced by 10 mL of fresh DMEM containing the nanoemulsions, followed by incubation for 4 h. The cells were trypsinized and collected by centrifugation, and then fixed using 4% formaldehyde at 4 °C overnight. The cells were then mixed with 1% agarose gel in a 1.5 mL tube and visualized on a micro-CT (Simens Inveon Micro-CT, Siemens Medical Solutions, Knoxville, TN, USA).

### Cellular uptake of fluorescently labeled Nanoemulsions

To probe the cellular uptake of lipiodol nanoemulsions, 1.64 wt-% of Nile red was incorporated in the core of the nanoemulsions. HeLa cells were seeded in a chamber slide (4-well Culture Slides, BD Falcon, MA) at a density of 10^5^ cells per well, and pre-incubated in DMEM at 37 °C for 24 h. The medium was then replaced by Nile red-loaded nanoemulsions at a concentration of 1.2 μg mL^−1^ in a fresh medium, and the cells were treated for 4 h at 37 °C. Afterwards, the nanoemulsions were removed, and the cells were washed several times with PBS. Then the cells were fixed using 4 wt-% formaldehyde for 30 min at room temperature. The nuclei of cells were stained using DAPI (1.5 mg mL^−1^) for 2 min, and the specimens were examined using a confocal laser scanning microscope (LSM510, Carl Zeiss, Oberkochen, Germany). Confocal images were quantitatively analyzed using ImageJ (U. S. National Institutes of Health, Bethesda, Maryland, USA).

### Cytotoxicity

HeLa cells were cultured in DMEM (10% FBS and 1*%* penicillin/streptomycin) at 37 °C. The cells were seeded onto a 96-well-plate at a density of 10^4^ cells per well, followed by incubation at 37 °C for 24 h. The medium was replaced by a fresh DMEM containing pNEs at various concentrations. The cells were then incubated for 4 h, and the pNEs were removed from the culture medium. The cells were further incubated for 2 days in a fresh medium, and then treated with a CCK-8 assay solution. The absorbance at 450 nm was recorded using a microplate reader (CLARIO Star, BMG Labtech, Germany).

## Results

**Fig. 1:**
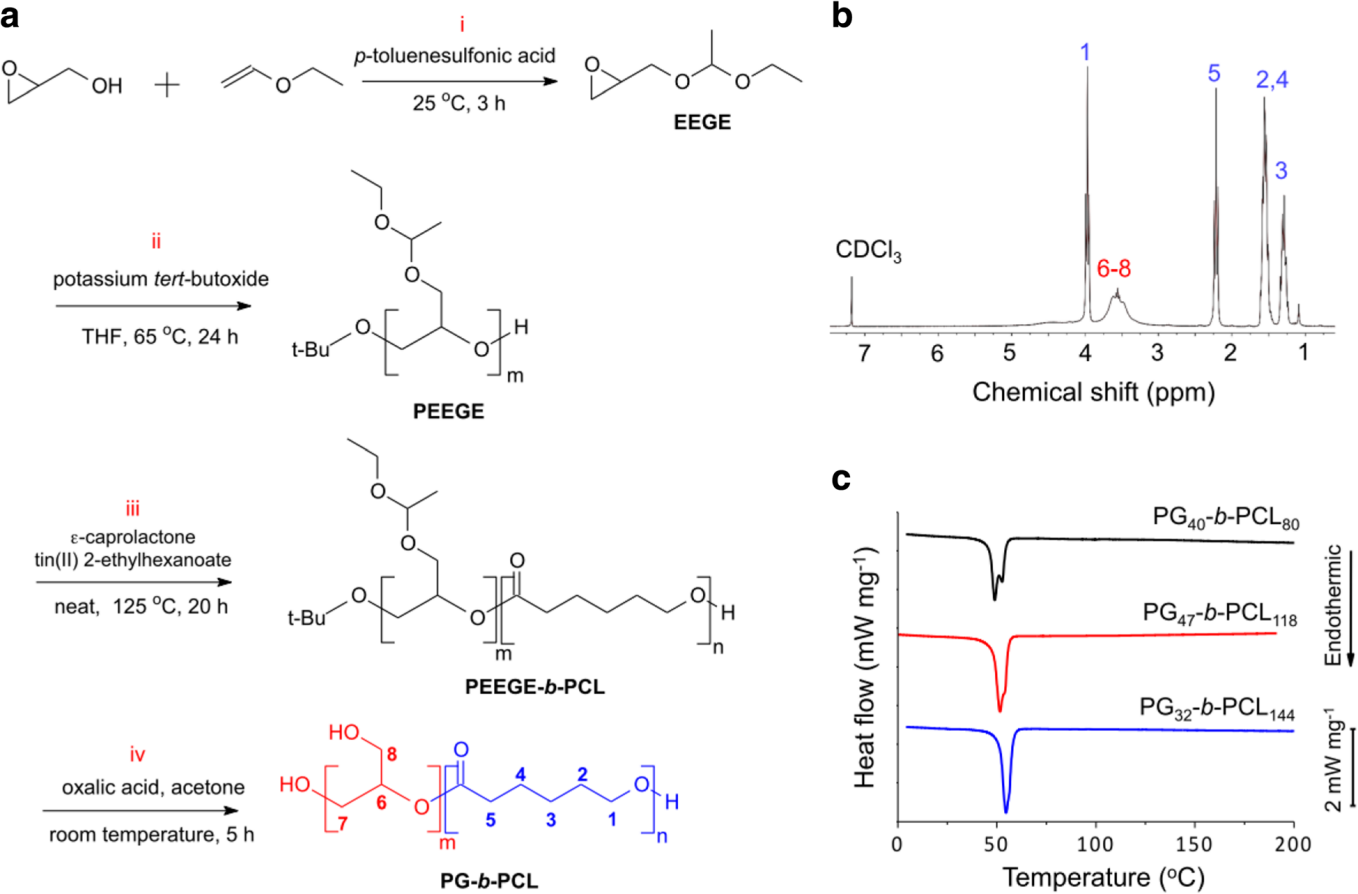
Synthesis and characterization of PG-*b*-PCL: **a** Synthetic scheme of PG-*b*-PCL: (i) Synthesis of EEGE, (ii) Polymerization of PEEGE, (iii) Ring-opening polymerization of ɛ-caprolactone to PEEGE-*b*-PCL, and (iv) De-protection of PEEGE to synthesize PG-*b*-PCL; **b** ^1^H NMR spectrum of PG_40_-*b*-PCL_80_ with the corresponding functional peaks shown in the chemical structure of PG-*b*-PCL in (**a**); **c** DSC diagram of PG-*b*-PCL in the second heating scan

The synthetic scheme of PG-*b*-PCL with a linear block of PG is summarized in Fig. 1a. The hydroxyl group of glycidol was temporarily protected through the reaction of glycidol with ethyl vinyl ether. The resulting EEGE was then polymerized by ring-opening polymerization to PEEGE. The synthesized PEEGE was used as an initiator for the ring-opening polymerization of ε-caprolactone to PEEGE-*b*-PCL. The hydroxyl groups of PEEGE-*b*-PCL were finally de-protected to obtain linear PG-*b*-PCL. The chemical structure of the synthesized block copolymer was analyzed using ^1^H NMR (Fig. 1b). Peaks number ‘1’ to ‘5’ in the spectrum were assigned to the hydrogen atoms in PCL, and peaks number ‘6’ to ‘8’ to PG. The integrations of the corresponding peaks in the NMR spectrum were analyzed to calculate the molar ratio of the two blocks, as shown in Table 1. Three compositions of PG-*b*-PCL with different molar ratios of the two blocks were selected for further investigation: the molar ratios of PCL to PG were 2, 2.5, and 4.5, and the corresponding polymers are denoted as PG_40_-*b*-PCL_80,_ PG_47_-*b*-PCL_118_, and PG_32_-*b*-PCL_144_ (Additional file 1: Figure S1). The subscripts represent the number of repeating units calculated from the molecular weight and the molar ratios. The number average molecular weights (M_n_) were in the range of 6–12 kDa, and the weight average molecular weights (M_w_) were in the range of 12–19 kDa. The polydispersity indexes were from 1.5 to 1.9. The DSC diagrams of the three copolymers are shown in Fig. 1c. The melting temperatures (49, 52, and 55 °C) and crystallinity (enthalpy = 58, 71, and 67 J g^-1^) of the copolymers increased as the length of the PCL increased, which might have been caused by the crystallization of the PCL chain.

**Table 1:** Molecular weights of PG-*b*-PCL with different lengths of PG and PCL

Polymer	M_n_^a^	M_w_^a^	PDI^b^	Molar ratio of PG:PCL^c^
PG_40_-*b*-PCL_80_	6246	12,204	1.95	1: 2
PG_47_-*b*-PCL_118_	11,250	16,989	1.51	1: 2.5
PG_32_-*b*-PCL_144_	12,258	18,822	1.53	1: 4.5

^a^Determined by GPC

^b^Polydispersity index = M_w_/M_n_

^c^Determined using ^1^H NMR

**Fig. 2:**
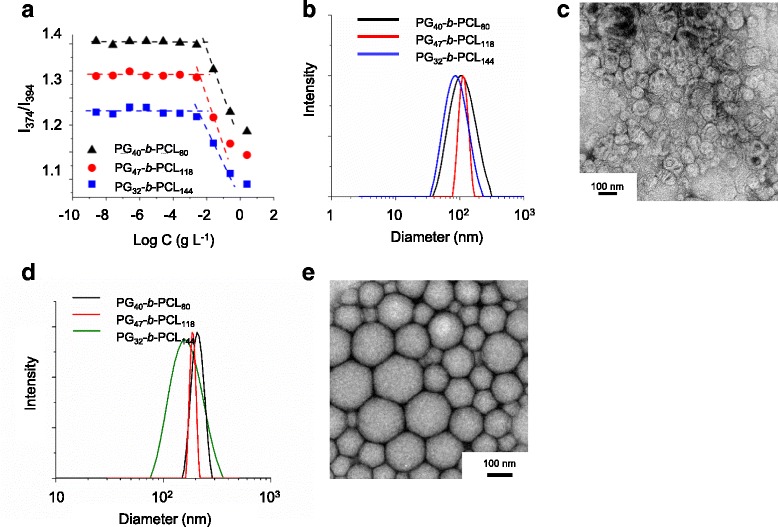
Characterization of polymeric micelles: **a** Fluorescent intensity ratio of pyrene as a function of PG-*b*-PCL concentrations. Hydrodynamic size distribution **b** and TEM image **c** of bMCs. Hydrodynamic size distribution **d** and TEM image **e** of bNEs

Polymeric micelles (denoted as ‘bMCs’) were prepared using PG-*b*-PCL with different lengths of the PCL block, and their critical aggregate concentrations (c.a.c.) were determined using pyrene. As expected, the c.a.c. value of PG_40_-*b*-PCL_80_ (7.4 × 10^−3^ g L^-1^) was much higher than that of PG_47_-*b*-PCL_118_ and (4.0 × 10^−3^ g L^−1^), and PG_32_-*b*-PCL_144_ (3.5 × 10^−3^ g L^-1^) (Fig. 2a). This is because the relatively larger segment of PCL decreased c.a.c. due to its increased hydrophobicity. However, the size distribution of bMCs of PG_32_-*b*-PCL_144_ and PG_40_-*b*-PCL_80_ was significantly broader than that of PG_47_-*b*-PCL_118_ (the hydrodynamic diameter = 113.9 nm, PDI = 0.11) (Fig. 2b). The bMCs exhibit a relatively uniform spherical morphology with a diameter of 83 ± 29 nm (Fig. 2c). The smaller particle size from TEM might be due to the shrinkage during the drying process. Figure 2d and e show the size distributions and TEM image of blank lipiodol nanoemulsions (bNEs). The result indicates that bNE-1, which was prepared using 10 mg PG_47_-*b*-PCL_118_ and 50 mg of lipiodol by a stirring method, had the smallest diameter (181 nm). Considering the physiochemical characteristics of self-assembled aggregates, we chose PG_47_-*b*-PCL_118_ for further investigation on the preparation of nanoemulsions incorporating lipiodol as a core using various emulsification parameters listed in Table 2. The loading yield and efficiency of lipiodol were 75.5 and 90.6%, respectively, as determined using the calibration curve from the absorption at 260 nm (Additional file 1: Figure S2).

**Table 2:** Experimental parameters for the preparation of bNEs, mean hydrodynamic diameter, and polydispersity index

Batch No.	m_1_ (mg)^a^	m_2_ (mg)^b^	V_acetone_ (mL)	Emulsification condition			Mean Diameter (nm)^c^	PDI^d^
				Stirring speed (rpm)	Temperature (°C)	Method		
bNE-1	10	50	3	1000	45	Magnetic stirring	181.0	0.128
bNE-2	10	100	3	1000	45	Magnetic stirring	215.0	0.081
bNE-3	10	150	3	1000	45	Magnetic stirring	231.8	0.101
bNE-4	10	100	1	500-700	25	Magnetic stirring	341.1	0.190
bNE-5	10	100	1	500-700	45	Magnetic stirring	233.2	0.085
bNE-6	10	100	1	1000	25	Magnetic stirring	219.3	0.087
bNE-7	10	100	3	500-700	25	Magnetic stirring	226.9	0.119
bNE-8	10	100	3	10,000	25	Homogenization	286.2	0.139
bNE-9	10	100	3	20,000	25	Homogenization	349.0	0.171
bNE-10	10	100	3	30,000	25	Homogenization	264.4	0.194
bNE-11	10	100	3	10,000	45	Homogenization	250.1	0.096

^a^m1 = the mass of PG-*b*-PCL

^b^m2 = the mass of lipiodol

^c^Determined by DLS

^d^Polydispersity index = *μ*_*2*_ */Γ* ^*2*^, estimated by the cumulant method

**Fig. 3:**
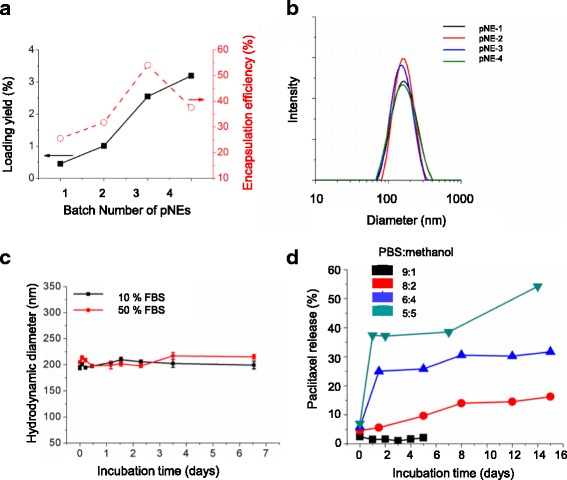
Preparation and characterization of paclitaxel loaded nanoemulsion (pNE): **a** Loading yield and encapsulation efficiency of paclitaxel within lipiodol nanoemulsions. **b** Size distribution of pNEs. **c** Dispersion stability of pNEs in PBS containing 10 and 50% FBS at 37 °C. **d** In vitro release profiles of paclitaxel from pNEs in a mixture of PBS and methanol

Nanoemulsions with various concentrations of paclitaxel were prepared using PG_47_-*b*-PCL_118_, as described in Table 3, and the loading yield and efficiency were determined. The nanoemulsions fed with 3 mg (2.6%) of paclitaxel (pNE-3) showed the highest encapsulation efficiency (55%) (Fig. 3a). The lower efficiency at a higher loading yield might be caused by the limited solubility of paclitaxel in lipiodol, which is about 10 mg mL^−1^. Despite the different loading yield of paclitaxel, no significant difference in the size distribution of pNEs was observed (Fig. 3b). To evaluate the PG-*b*-PCL nanoemulsions as drug delivery vehicles, we examined the dispersion stability of pNE-3 in 10 and 50% FBS. No significant change of their hydrodynamic diameter was observed for a week at 37 °C (Fig. 3c). This is attributed to excellent biocompatibility and the high resistance of PG to non-specific protein adsorption.

**Table 3:** Experimental parameters for pNE, mean hydrodynamic diameter, and polydispersity index

Batch No.	Weight PG_47_-*b*-PCL_118_ (mg)	m_Lipiodol_ (mg)	m_Paclitaxel_ (mg)	V_acetone_ (ml)	Mean Diameter^a^ (nm)	PDI^b^
pNE-1	12.3	59.8	1.2	3	174.0	0.090
pNE-2	12.0	56.4	2.1	3	166.2	0.063
pNE-3	12.3	56.5	3.3	3	158.9	0.065
pNE-4	10.6	51.4	5.1	3	171.1	0.094

^a^Determined by DLS

^b^Polydispersity index (PDI) = *μ*_*2*_/*Γ* ^*2*^, estimated by the cumulant method

Emulsification condition of every batch is the same with that of  bNE-1 (magnetic stirring at 10,000 rpm at 40–50 °C)

Next, we determined the in vitro release profiles of paclitaxel from pNEs (pNE-3). Due to the negligible solubility of paclitaxel in an aqueous solution, surfactants or polar organic solvents are commonly added to the release medium to maintain the sinking condition. However, there is no standardized experimental condition presumably because of the differences in the properties of materials used for paclitaxel formulations. In this work, a mixture of PBS and methanol was used to examine how solvent polarity affects the release of paclitaxel from pNEs (Fig. 3d). The sink condition was maintained if the volume percentage of methanol was higher than 20%. In 50% methanol, 37.4% of paclitaxel was released from pNEs for 24 h at the initial stage. After 24 h, the release rate became lower, and for 15 days only 54.3% of the paclitaxel was released. The drug release is controlled by slow diffusion of paclitaxel as a result of partitioning from the hydrophobic core of the nanoemulsions into 40 and 50% methanol during the first day, followed by slow release. However, in 20% methanol, the drug release was very limited to less than 20% for 2 weeks, and no significant release of pactlitaxel was observed in 10% methanol. The results indicate that the hindered diffusion of paclitaxel through the PCL-based interphase is a limiting factor for the diffusion of paclitaxel from the lipiodol core to the release medium.

To achieve active targeting via FA receptor (FAR)-mediated endocytosis, pNEs were conjugated with FA by boronic acid ester linkage to produce fpNEs as described in the experimental section. Boronic acid binds to alcohol with high affinity through the formation of boronate ester, which is a strong single-pair reversible functional group, interaction in an aqueous milieu [10]. FBA was synthesized through simple amide coupling between FA and 3-aminophenylboronic acid. Boronic acid can simply react with the vicinal diol groups at the end of the PG block to form double ester linkages with each boronate unit with a degree of folate substitution (19.2%).

**Fig. 4:**
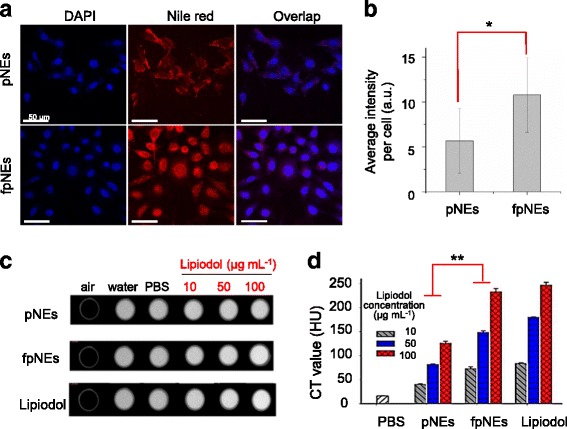
Enhanced intracellular uptake of folate deocorated nanoemulsions (fNEs): **a** Confocal fluorescence microscopic images of HeLa cells incubated with Nile red-labeled nanoemulsions with and without FA conjugation. Scale bars = 50 μm. **b** The average intensity of individual cells (*p = 0.001). Micro-CT images **c** and HU values **d** of phantoms of HeLa cells treated with deionized water, PBS, pg-pNE, fpg-pNE, and lipiodol (*n* = 3, Error bars indicate standard deviation, **p < 0.05)

To visualize the cellular uptake of pNEs, Nile red was incorporated as a hydrophobic fluorescent dye into the nanoemulsions. HeLa cells, which overexpress FAR type α, were incubated with the nanoemulsions loaded with 1.64 wt-% Nile red, and examined using a confocal fluorescence microscope (Fig. 4a). The Nile red-loaded nanoemulsions are shown in red, while DAPI staining showed the area and distribution of the nuclei. The fluorescence intensity per individual cell is represented in Fig. 4b. The average intensity per single cell was almost twice higher in the cells treated with fpNEs than those treated with pNEs. These results indicated that the FA conjugation efficiently increased the cellular uptake of the nanoemulsions presumably through binding to FARs in the cellular membrane. Moreover, assuming that the density of PG-*b*-PCL is 1.1 g mL^−1^, FA ligands were located on the surface of nanoemulsions at a density of 6.8 FA molecules per nm^2^ [31]. It can be inferred that the high density of FA on the nanoemulsions enhanced the cellular uptake efficiency by multivalent binding to HeLa cells. It was also confirmed by competition assay of folate receptor mediated intracellular uptake of fpNEs. In case that folate receptors are blocked by cultivating cells in folic acid-rich medium, the intracellular uptake efficiency of fpNEs decreased to 30.7% of folate receptor open system (Additional file 1: Figure S3).

Lipiodol within the nanoemulsions can serve as a CT contrast agent, so the diagnostic capability of the lipiodol nanoemulsions was evaluated using micro-CT analysis. HeLa cells were treated with PBS, pNEs, and fpNEs, followed by the fixation in an agarose gel phantom (Fig. 4c). The emulsions concentration was varied to achieve from 10 to 100 μg mL^−1^ of lipiodol. The CT values of the cells (1 × 10^7^) treated with fpNEs and pNEs, where the concentration of lipoidol was 10 μg mL^−1^, were 73.4 ± 3.6 and 40.3 ± 1.4 HU, respectively, where HU is a hounsfield unit (Fig. 4d). The CT value increased with increasing the concentration of lipiodol. The CT values of cells treated with pNEs were 82.0 ± 0.9 and 126.4 ± 3.8 HU at 50 and 100 μg mL^−1^ of lipiodol, respectively. The values of cells treated with fpNEs were 148.7 ± 3.1 and 233.4 ± 6.4 HU at 50 and 100 μg mL^−1^ of lipiodol, respectively. These values were much higher than those from treatment with pNEs. The CT values of most soft tissues are in the range of 30–100 HU, and the difference of 50–100 HU can be used to distinguish the tissues of interest. Therefore, it can be said that fpNEs are applicable as contrast agents for CT imaging of tumors. Interestingly, regardless of the lipiodol concentration, the CT value of fpNEs was about 1.8 times higher than that of pNEs, which corresponds to the difference in the fluorescence intensity per cell. The difference seems to result from the cellular uptake efficiency of nanoemulsions. Compared with our previous study on the feasibility of CT imaging using lipiodol nanoemulsions [30] (therein, denoted ‘*sr*-NEs’) composed of LPEI-*g*-cholesterol, cholesterol, DSPE-PEG, siRNAs, and paclitaxel, the contrast enhancement by fpNEs was 1.8-fold better than that from the use of *sr*-NEs. This was true even with the lesser amount of lipiodol (ca. 72% of fpNEs), probably due to difference in the cellular uptake. Thus, it is expected that fpNEs could be further developed as an in vivo tumor-targeting CT contrast agent.

**Fig. 5:**
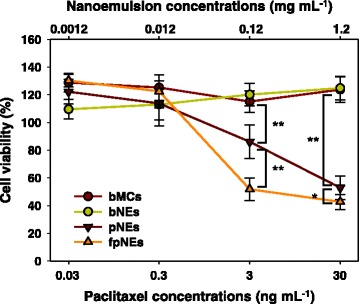
Cytotoxicity to HeLa cells of nanoemulsions with and without FA conjugation with different concentrations of paclitaxel. bMCs and bNEs were used as negative controls (*p < 0.1, **p < 0.01)

The effects of FA conjugation on the cytotoxicity of paclitaxel-loaded lipiodol nanoemulsions were examined using HeLa cells (Fig. 5). bMCs and bNEs were also used as negative controls to determine the cytotoxicity of PG_47_-*b*-PCL_118_ and lipiodol. The bMCs and bNEs exhibited no significant cytotoxicity up to a micelle concentration of 1.2 μg mL^−1^. In contrast, both pNEs and fpNEs caused efficient death of target cells. At 1.2 μg mL^−1^ of nanoemulsions, pNEs showed about 53% cell viability on HeLa cells at a paclitaxel concentration of 30 ng mL^−1^, while fpNEs showed 41% cell viability at the same concentration. However, at 3 ng mL^−1^, fpNEs exhibited 52% cell viability while pNEs had 85%. The result indicates that the FA conjugation to the surface of lipiodol nanoemulsions effectively promotes the intracellular delivery of the nanocarriers to boost the efficacy of paclitaxel.

## Discussion

Although most widely used as a hydrophilic corona of polymeric nanoparticles for biomedical applications, PEG has several limitations such as lowering cellular uptake of nanoparticles, a lack of responsiveness to environmental changes, and insufficient number of sites for chemical modification. Hence, a development of a novel hydrophilic moiety is highly demanded. PG is one of the most prominent alternatives to PEG due to tis excellent dispersion stability and biocompatibility. We synthesized PG-*b*-PCL amphiphilic diblock copolymers having critical aggregation concentrations in the range of 10^−6^ - 10^−7^ M which is comparable to that of PEG based polymeric emulsifiers. Theranostic nanoemulsions co-encapsulating lipiodol and paclitaxel were prepared using PG_47_-*b*-PCL_118_.

The potency of our theranostic nanoemulsions is attributed to the high loading yield of paclitaxel in lipiodol core and the high degree of cell-targeting ligand on the PG corona. As reported previously, to achieve efficient drug encapsulation, it is critical to select a suitable surrounding medium which has a high miscibility and solubility to the payloads. Besides serving as a CT contrast agent, lipiodol which has paclitaxel solubility of 10 mg mL^−1^ was a good medium for encapsulated paclitaxel. Moreover, lipiodol played a role for a reservoir of paclitaxel by hindering diffusion of paclitaxel from the core through the PCL-based interphase. Considering that small molecular payloads of nanoparticles are easily diffused out during blood circulation and found in plasma or accumulated in non-target-tissues like kidney, this encapsulation stability resulted from lipiodol core can be one of the outstanding advantage for the delivery efficiency in vivo.

In addition, multiple hydroxyl groups on the PG’s backbone enable to introduce a high degree of hydrophobic cancer-targeting ligand on the surface of nanoemulsions without a loss of dispersion stability in aqueous. The conjugation strategy between the vicinal diol of PG and the folate boronic acid (FBA) addressed the laborious work for introducing targeting moiety to nanoemulsions and simplified the process by enabling post-modification of nanoemulsion surface in aqueous media. The folate decorated nanoemulsions increased intracellular uptake by HeLa cells via receptor-mediated endocytosis, which consequently led to enhancement of CT imaging and anticancer therapeutic effect.

Although the theranostic efficacy of PG-PCL lipiodol nanoemulsion was only evaluated by in vitro assay in this study, it is expected that this nanoemulsion system could be further developed as an in vivo tumor-targeting theranostic contrast agent.

## Conclusion

This study demonstrated that PG-*b*-PCL diblock copolymers can serve as a semi-solid polymeric emulsifier for theranostic nanoemulsions for cancer. The PCL block can generate a robust polymeric layer at the oil/water interface because it is not soluble in lipiodol. The PG block has a hydroxyl group in each monomeric unit, exhibiting excellent biocompatibility and anti-fouling property. In vitro cell studies with HeLa cells demonstrated that ligand conjugation efficiently increased the cellular uptake of PG-*b*-PCL nanoemulsions. The FA-conjugated lipiodol nanoemulsions loaded with 2.6% paclitaxel showed excellent anti-cancer activity (LD_50_ < 3 ng mL^−1^), while placebo nanoemulsions did not show any significant cytotoxicity at the same concentration. In addition, the lipiodol nanoemulsions greatly enhanced the CT contrast of tissue-mimicking phantoms of HeLa cells due to efficient intracellular translocation. This study suggests that PG-*b*-PCL can serve as a robust polymeric emulsifier to stabilize nanoemulsions while enabling facile surface functionalization with multiple targeting molecules for targeted delivery of therapeutic and imaging agents.

## Additional file

Additional file 1: Supporting information. ^1^H NMR of PG-*b*-PCL, UV-Vis absorption spectra of lipiodol nanoemulsions, and confocal fluorescence microscopic images of HeLa cells. (DOCX 960 kb)

## Abbreviations

bMCs: Placebo polymeric micelles
bNE: Placebo nanoemulsions
CT: Computer tomography
EEGE: Ethoxyethyl glycerol ether
FA: Folic acid
FBA: Folate boronic acid
fpNE: Folate-decorated paclitaxel-loaded nanoemulsion
PCL: Poly(ε-caprolactone)
PEEGE: Poly(ethoxyethyl glycerol ether)
PEG: Polyethylene glycol
PG: Polyglycerol
PG-*b*-PCL: Linear polyglycerol-poly(ε-caparolactone) diblock copolymer
