# Supplementary material for: Lipiodol nanoemulsions stabilized with polyglycerol-polycaprolactone block copolymers for theranostic applications
Source: Biomater Res. 2017 Oct 17;21:21. doi: 10.1186/s40824-017-0108-4 (PMC5645845; doi:10.1186/s40824-017-0108-4)
Supplement: Additional file 1: — Supporting information. 1H NMR of PG-b-PCL, UV-Vis absorption spectra of lipiodol nanoemulsions, and confocal fluorescence microscopic images of HeLa cells. (DOCX 960 kb) [file 40824_2017_108_MOESM1_ESM.docx]

**Supporting Information**

**Lipiodol Nanoemulsions Stabilized with PG-PCL Block Copolymers for Theranostic Applications**

**Trang Huyen Le Kim^1^, Hwiseok Jun^1^, Jin Ho Kim^2^, Keunchil Park^2,3^,**

**, Jee Seon Kim^1,*^, and Yoon Sung Nam^1,4,*^**

^1^Department of Materials Science and Engineering

Korea Advanced Institute of Science and Technology,

291 Daehak-ro, Yuseong-gu, Daejeon, 34141, Republic of Korea

^2^Samsung Medical Center

Samsung Biomedical Research Institute

Irwon-dong, Gangnam-gu, Seoul, 06351, Republic of Korea

^3^Division of Hematology and Oncology, Department of Medicine,

Samsung Medical Center, Sungkyunkwan University School of Medicine,

Irwon-dong, Gangnam-gu, Seoul, 06351, Republic of Korea

^4^KAIST Institute for the NanoCentury

Korea Advanced Institute of Science and Technology,

291 Daehak-ro, Yuseong-gu, Daejeon, 34141, Republic of Korea

* To whom correspondence should be addressed.

Email: eliekim@kaist.ac.kr (J.S.K.) and [yoonsung@kaist.ac.kr](mailto:yoonsung@kaist.ac.kr) (Y.S.N.)

**Figure S1.** ^1^H NMR spectra of PG_40_-*b*-PCL_80_ (**a**), PG_47_-*b*-PCL_118_ (**b**), and PG_32_-*b*-PCL_144_ (**c**).

**
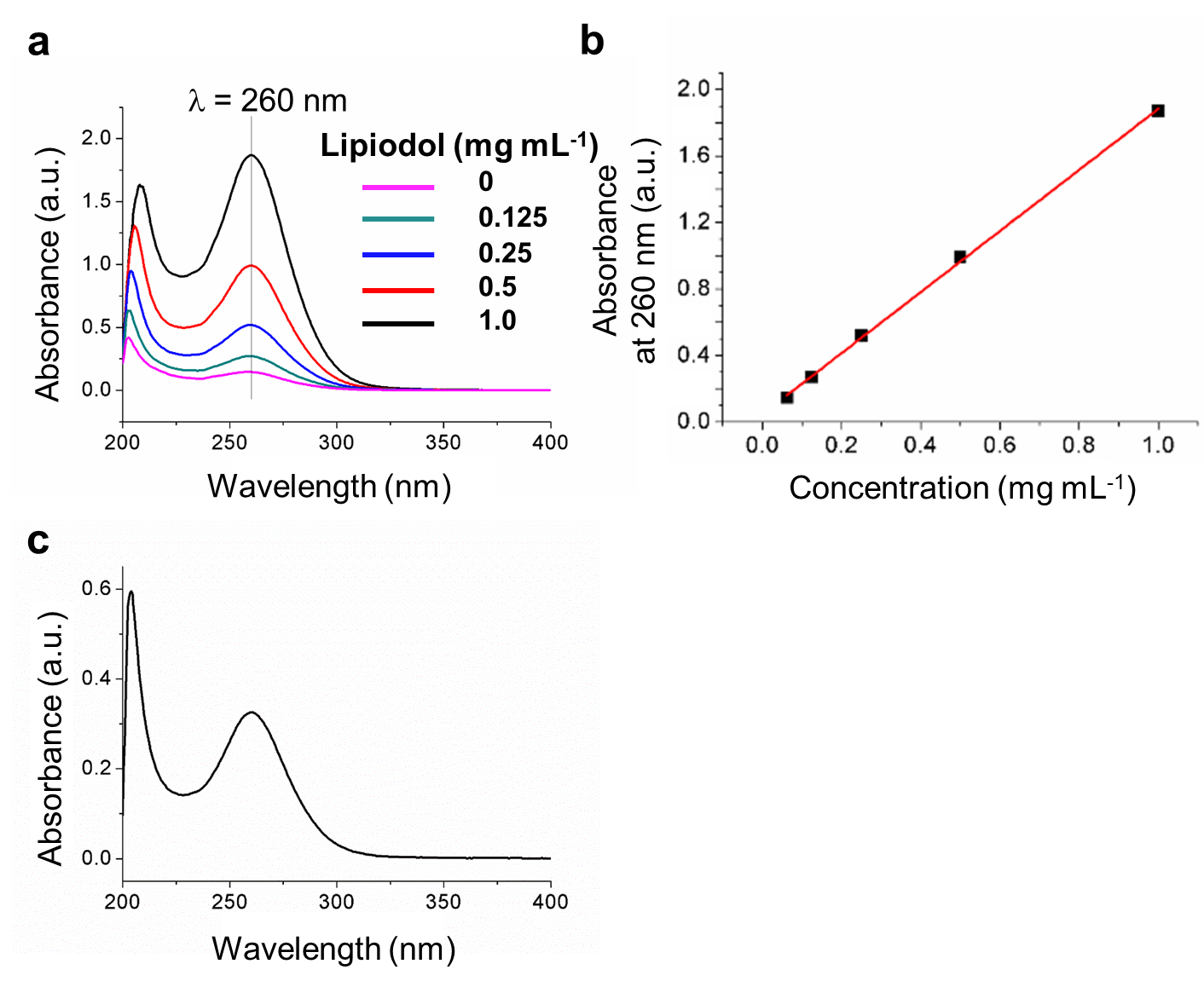
**

**Figure S2.** (**a**) UV-Vis absorption spectra of lipiodol in ethanol at concentrations from 0 to 1 mg mL^-1^. (**b**) A calibration curve for lipiodol. (**c**) UV-Vis absorption spectrum of lipiodol nanoemulsions.

**
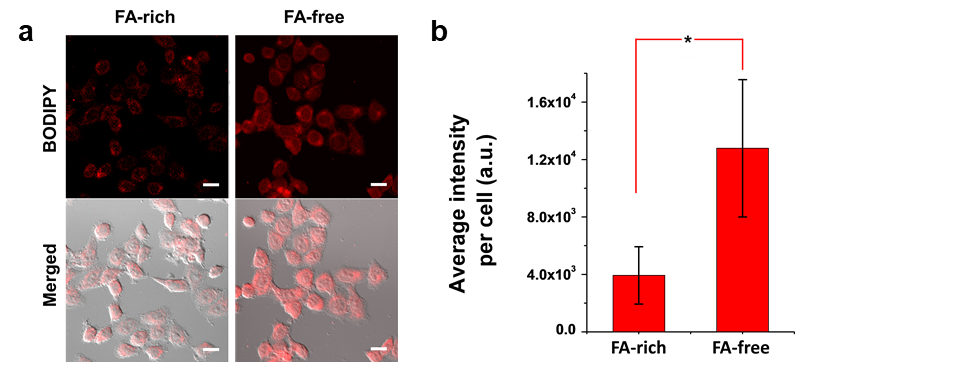
**

***Figure S3. (a) Confocal fluorescence microscopic images of HeLa cells incubated with fpNEs incorporating BODIPY-paclitaxel in folic acid-rich and folic acid-free medium. Scale bars = 20 μm. (b) The average intensity of individual cells (*p < 0.05).***
